# Disposal of Unused and Expired Medicines within the Sunyani Municipality of Ghana: A Cross-Sectional Survey

**DOI:** 10.1155/2022/6113346

**Published:** 2022-05-26

**Authors:** Ivy Anima Amoabeng, Bernice Araba Otoo, Godfred Darko, Lawrence Sheringham Borquaye

**Affiliations:** ^1^Department of Chemistry, Kwame Nkrumah University of Science and Technology, Kumasi, Ghana; ^2^Central Laboratory, Kwame Nkrumah University of Science and Technology, Kumasi, Ghana

## Abstract

The occurrence of pharmaceuticals in the environment is a global challenge. Pharmaceuticals such as antibiotics and analgesics have been reported in various environmental matrices at varying concentrations. The major disposal route for unused and expired pharmaceuticals in Ghana is throwing them into dustbins. Although there are laws on the proper disposal patterns of drugs, these laws are poorly implemented. Sunyani is a fast-growing community with several health facilities that dispense medications daily. The purchase and use of medications among households are also high. However, no data exist on the disposal patterns of pharmaceuticals within the Sunyani Municipality. This study aims to identify the disposal patterns for unused and expired medications by households and pharmacies within the Sunyani Municipality, Ghana. A descriptive cross-sectional study was conducted among 400 persons in homes and 35 persons from randomly selected pharmacies and over-the-counter medication shops (OTCMs) within the Sunyani Municipality. A face-to-face interview approach using structured questionnaires for each respondent was employed. Household respondents disposed of unused and expired medications mainly through dumping in garbage cans (70.8%), incineration (11.5%), and flushing down the sink (9%). Pharmaceutical shop respondents also disposed of unused and expired medications into dump cans, by incineration, through the Food and Drugs Authority of Ghana, and took back to pharmaceutical wholesalers. Disposal practices observed in this study by households and pharmacy respondents were largely inappropriate. This could be due to the lack of education on the proper disposal patterns available to these respondents. It is recommended that guidelines on safe disposal be put in place, and a structured procedure for collecting unused and expired pharmaceuticals should be introduced.

## 1. Introduction

Pharmaceutically active compounds are used widely to prevent or treat diseases in humans and animals and are usually classified based on their various therapeutic uses [1]. Two classes of pharmaceuticals often in high demand are analgesics (painkillers) and antibiotics. The increased demand for these drugs results from their use in treating common illnesses and infections (such as headaches, common cold, and boils), their cheap cost, and their easy complementary nature with other drugs [2]. Recently, studies have indicated that these drugs are becoming ubiquitous in the environment. Various pharmaceuticals and their metabolites have been detected in surface [3, 4], ground [5], waste [6], drinking water [7], and in food [8, 9] as well as agricultural [10] and dumpsite [11] soils at varying concentrations from ng/L to mg/L. The presence of these pollutants in the environment causes adverse effects. These effects include aquatic toxicity, endocrine disruption in humans, livestock, and aquatic life, and the development of antibiotic resistance [12].

The occurrence of pharmaceutical residues in the environment has been mainly attributed to anthropogenic activities. These activities include the release of untreated wastewater from hospitals, clinics, and pharmaceutical companies, the usage of sewage sludge from waste treatment plants as fertilizer on farmlands, the usage of untreated wastewater for crop irrigation, and the indiscriminate disposal of unused and expired pharmaceuticals directly into the external environment [13]. In Ghana, respondents from a survey conducted at Bohyen and Kaase, two suburbs in Kumasi, Ashanti region, corroborated that household trash, sinks, and incinerators were the frequently used routes for the disposal of unused and expired medicines [14]. The absence of comprehensive drug disposal schemes played a major factor in the choice of the disposal route used by respondents in this area. The lack of legislation, implementation, and education on the proper disposal of unused and expired drugs was the major contributor to the inappropriate disposal of unused and expired medicines.

In the USA, the Environmental Protection Agency under the Public Law 103–399 is the regulatory body in charge of the proper disposal of pharmaceutical waste and industrial wastewaters. The US Food and Drugs Authority (FDA) in agreement with local pharmaceutical stores and the local police department also regulate the proper disposal of unused and expired drugs. These agencies advise and dispose of unused and expired medications by flushing them down the sewage (medicines on the FDA flush list), take-back programs (to local pharmacies and over-the-counter medication shops (OTCMs)), and incineration. However, the most preferred method of disposal for these bodies is through the use of take-back programs and the implementation of a Disposal of Unused Medicine Properly (DUMP) project. In this project, leftover and expired drugs are collected by pharmacies and OTCMs and delivered to manufacturers or appropriate authorities for proper disposal [15].

In Ghana, the National Drug Policy stipulates that the Food and Drugs Authority (FDA) shall ensure that in collaboration with other agencies where appropriate, suitable measures are instituted for the regular identification, collection, and safe disposal of expired drugs and drug waste [16]. The FDA of Ghana, under Section 148 of the Public Health Act, 2012 (Act 851), is obligated to supervise the safe disposal of regulated products that are unwholesome for both human and animal consumption. The FDA, however, does not have policies that cater specifically for the safe disposal of unused and expired drugs.

Given the challenges with most of these policies, the most employed disposal method for unused medications is dumping into trash cans. The practice of flushing drugs into the sewage system still takes place in a few other countries [17]. The occurrence of pharmaceuticals in the environment can only be minimized if systems are put in place to dispose of unused and expired drugs properly. At present, little or no data are available on how households, pharmacies, and even organizations dispose of unused and expired portions of medications in most regions in Ghana.

Sunyani, the capital of the Bono region, is a municipality with a population that increases by 3.8% per annum [18]. An increase in a population usually correlates with an increase in the purchase, use, and possible abuse of drugs. In Sunyani, most hospitals, pharmacies, over-the-counter medication shops (OTCMs), and even households have no regulations to dispose of unused and expired medicines. Most individuals and institutions predominately dump these pollutants in trash cans, flush them down the toilet, or incinerate them. This leads to their potential environmental occurrence, persistence over time, bioaccumulation, adverse effects on some nontarget organisms, and the potential buildup of drug resistance in the environment. This study evaluated the disposal patterns of unused and expired medicines by households, pharmacies, and OTCMs in the Sunyani Municipality. Respondents' awareness of environmental issues associated with improper disposal of their medicines and their willingness to support a state-run disposal and destruction system for unused and expired medicines were also explored.

## 2. Methods

### 2.1. Study Area

The survey was conducted in the Sunyani Municipality, the capital of the Bono Region, Ghana, West Africa. According to the 2017 population census, the Sunyani Municipality has about 152,567 persons and a population growth of about 3.8% per annum [18]. An increase in demand for medicines accompanies the rapid increase in population. The presence of hospitals, clinics, pharmacies, and over-the-counter medication shops whose daily routine involves the dispensary and disposal of medicines makes this area a potential spot for unsafe disposal of unused portions of these drugs and, consequently, the occurrence of pharmaceuticals in the environment.

### 2.2. Survey Design

A cross-sectional survey was conducted in April 2021 within the Sunyani Municipality. Data collection lasted for two weeks.

### 2.3. Sample Size Determination and Sampling

Andrew Fisher's formula (equation shown below) was used to determine the sample size of household respondents in this study.

(1) $$\begin{array}{c} \text{Sample size}=\frac{{\left(z-\text{score}\right)}^{2}\times \text{StdDev}\times \left(1-\text{StdDev}\right)}{\left(\text{confidence interval}\right)2}. \end{array}$$

To calculate the sample size of household respondents, the population size of the Sunyani Municipality was first obtained from the Ghana statistical service as 152,567 persons as per the 2017 population census. The confidence level and interval were also determined as 95% and ±4.90%, respectively. A standard deviation of 0.5 was chosen with a *z*-score of 1.96. The sample size was computed as 400 using the formula. Four hundred household respondents were, thus, interviewed face-to-face, a participant each from a household. For each of the 8 suburbs in Sunyani, 50 households were selected. Households were selected based on willingness to participate in the study. Of about 41 pharmacies and OTCMs in the municipality, data were collected from 35 pharmacies and over-the-counter medication shops. The 35 pharmacies and OTCMs were selected because they were willing to participate in the study. The questionnaire was given to the pharmacists at various retail shops. Convenient sampling was therefore employed in this study.

### 2.4. Data Collection

Data were collected through a structured interviewer-administered questionnaire. The questionnaire elicited sociodemographic data, their disposal patterns of expired or unused medicines, and their knowledge of the environmental impact of inappropriate medication disposal. All the questions were close-ended, and some with an option for the respondents to record their own opinion if the choices were not suitable. The survey consisted of 18 and 15 structured questionnaires for households and pharmacies (Supplementary Materials).

### 2.5. Eligibility

Participation of respondents in this survey was voluntary. Participants were eligible to participate if they were 15 years old or above and had possessed unused or expired medicines in the past year. The participant should be a resident living within the Sunyani Municipality. The questionnaire was written in English, but when a participant did not fully understand English language, the interviewer verbally translated the questions into the local dialect (Twi) for the respondent. It was to ensure that respondents properly understood the questions being asked.

### 2.6. Data Processing and Analysis

Responses from the survey were carefully coded using numbers in Microsoft Excel and imported into the Statistical Package for the Social Sciences (SPSS 20) software for the final analysis. When participants left questions unanswered, it was treated as a missing value and not added to the survey results. Demographic variables of the survey (age, gender, educational level, and years of practice) were summarized with descriptive statistics of frequencies. The percentages of disposal practices employed in households and pharmacies were also summarized. Multiple response-single coding was employed to treat each item as continuous data.

### 2.7. Ethical Clearance and Informed Consent

All respondents signed the informed consent. The formal consent was also sought from guardians of all respondents younger than eighteen years. Ethical approval was obtained from the Ethics Committee for Research, KNUST.

### 2.8. Confidentiality

Respondents were assured that the data collected would remain confidential.

## 3. Results

### 3.1. Demographic Data for Households and Pharmacies

There was a 100% response rate in this study as all respondents approached agreed to participate. A total number of 400 selected respondents were used to represent households. Of this, 45.5% of the population was male and 54.5% was female. A simple majority of the respondents representing (54.3%) were aged between 15 and 30 years. Twenty percent of the respondents were pharmacists, 14.3% were technicians, 14.3% were pharmacy assistants, and 34.3% were over-the-counter drug sellers. The demographic data for both household and pharmacy respondents are given in Table 1.

### 3.2. Usage, Frequency, and Disposal of Unused and Expired Drugs in Households

**Figure 1:**
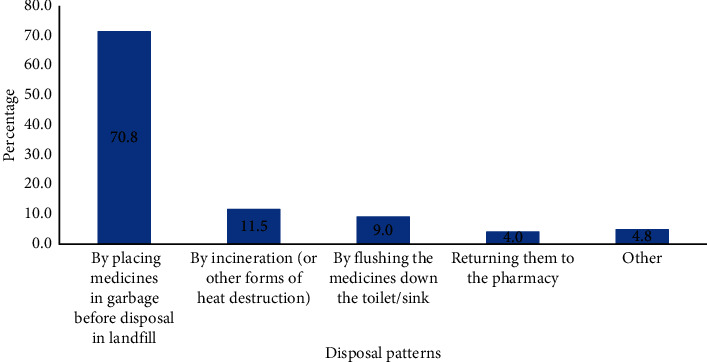
Disposal patterns of unused and expired drugs by household respondents.

Approximately 68% of respondents had medicines at home. 46.5% of the respondents had antibiotics at home, whereas 48.8% had analgesics at home. 54.5% of the respondents had leftover and expired medicines at home. The most frequently disposed drugs were antibiotics (29%) and analgesics (43%). Drugs were disposed of daily (5.8%), weekly (21.5%), and yearly (6.3%), and 43.8% of respondents disposed of medications when redundant. 283, representing 70.8% of the population, disposed of expired and unused medicines by dumping them in garbage cans. Content of these garbage cans eventually ends up on dumpsites.  Figure 1 shows household respondents' disposal patterns for unused and expired medication within the Sunyani Municipality.

### 3.3. Sales, Frequency, and Disposal of Unused and Expired Drugs in Pharmacies

**Figure 2:**
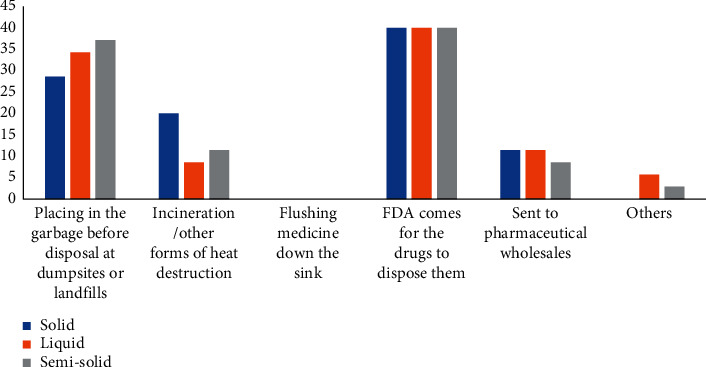
Disposal patterns of the frequent solid, liquid, and semisolid drugs by pharmacy respondents within the municipality.

The class of drugs frequently purchased from pharmaceutical respondents by persons living in the Sunyani Municipality was also assessed. Pain medications (analgesics) were the most purchased drug in the Sunyani Municipality with a 77% sales rate. For solid dosage forms of drugs such as tablets, capsules, suppositories, pessaries, and transdermal patches, 28.6% of the respondents indicated that they placed their unused or expired medicines in garbage before disposal at dumpsites or landfills. Twenty percent of the respondents incinerated these drugs or used other forms of heat destruction on them; 40% of respondents partnered with the FDA to dispose of these drugs.  Figure 2 shows the disposal patterns for solid, liquid, and semisolid dosages in a clustered chart.

### 3.4. Environmental Awareness of Household and Pharmacy Respondents on Pharmaceuticals as an Issue of Environmental Concern

As given in Table 2, the responses to questions intended to measure the literacy of the respondents on pharmaceuticals as an issue of environmental concern in the Sunyani Municipality. A total of 42.9% and 39.8% of pharmacy and household respondents, representing a slim majority, found take-back programs of leftover medicines to pharmacies and wholesales inconvenient. Most pharmacy respondents (62.9%) and household respondents (50.3%) were concerned about the natural environment. When asked if an environmental problem does not affect my health and property, I do not care; these respondents strongly disagreed.

## 4. Discussion

Many reports have presented findings that for most communities over the world, even though there are laws for the proper disposal of pharmaceuticals due to issues of cost and ineffectiveness in catering for both industrial and household pharmaceutical wastes, implementation of these laws are difficult [19–21]. The majority of household participants in the Sunyani metropolis affirmed having medications at home, with 46.5% of population having antibiotics at home and about 50% also having analgesics at home. This indicates the high usage of antibiotics and analgesics in the area. The prospective effect of increased use of these drugs within the Sunyani Municipality is the likely indiscriminate disposal of their leftover portions. Expired and unused medications were predominantly disposed of via garbage cans, which eventually ended up on dumpsites. This disposal method has been discussed as one of the major routes through which pharmaceuticals get into the environment [19, 22]. This establishes a pattern for the disposal of unused and expired medications in various households worldwide, with disposal through household trash being the most predominant.

The disposal of pharmaceuticals in household trash has become a universal disposal pattern for many institutions and households. In the USA, it is recommended for persons who find take-back programs inconvenient to mix unused and expired with an unpalatable substance such as coffee grounds. The mixture should then be placed in an enclosed container such as a sealed plastic bag and mixed with the regular household trash [23]. However, this method of disposal does not mitigate the impact pharmaceuticals have on the external environment. It is only a precautionary measure designed to reduce exposure to such drugs in other individuals, especially children. For drugs that are highly toxic and are on the flush list, it is recommended by the FDA that these drugs are flushed down the toilet into sewage systems [20]. Although this disposal method seems easy and safe, medicines such as diazepam and morphine sulfate can rust water pipes and block water drainage channels [23]. These medications being disposed of may end up in the lakes, streams, and even the community drinking water, affecting public health and aquatic life negatively. This is to say that although there are laws put in place worldwide to control the extent of damage by the presence of pharmaceuticals in the environment, they have proved futile in achieving the intended objectives.

In this study, most household respondents were unaware of pharmacy take-back programs, which indicated poor literacy on the proper disposal patterns for unused and expired drugs. Regulatory bodies such as the Ghana FDA must embark on an intense educational campaign to increase the general population's awareness of this option. Pharmacy shops should be mandated to inform patients of this option when they sell drugs to them. Although individuals were fully aware of pharmaceuticals as an issue of environmental concern, they believed that take-back programs to the pharmacies, if available, would be inconvenient. Respondents feared that due to the lack of enough storage for most local pharmacy shops in their vicinities, their leftover drugs might not be accepted, and they may have wasted their time and finances in the process. In view of this, most respondents preferred to avoid the stress and disappointment and instead dispose of their leftover pharmaceuticals as they please, randomly and carelessly. Regardless of all these aforementioned assertions, respondents agreed that preventing water pollution is essential even with no evidence. They cared deeply about environmental problems that were not necessarily about their health or property. Most of these respondents agreed to switch to environmentally friendly medications if the incumbent drug was not ecofriendly.

The influence of gender and age on household respondents' disposal of drug leftovers could not be related to this study. However, the age of the pharmacy and OTCM attendants may have influenced their response to the survey questions. The pharmacy respondents older than 45 years answered survey questions with a lot of experience as if they had worked at the job for a while. In a study by Abruquah in Kumasi [22], Ghana, no distinct correlation was made between age, gender, and disposal patterns. This result was also similar to another research conducted in the Kumasi Metropolitan Assembly [24]. Persons with higher educational backgrounds understood and showed much concern for the environment (Table 2). In a study conducted in Ireland (Cork and Galway), the city was thought to inhabit persons of higher educational achievements, with nearly 60% of the respondents having a third-level degree. In view of this, 23% of the respondents in Cork had heard of the DUMP campaign and 40% of the respondents had subsequently used the bins for disposal provided by the campaign [25]. In this study, a slight majority of the population of respondents had up to JHS education, which could impact these improper disposal patterns observed.

In the case of pharmacies, 35 respondents from the pharmacies represented the population. In terms of drug purchase and dispensary, mainly pain medications were dispensed, followed closely by antimalarial and antibiotics. These data showed that the dispensary of analgesics and antibiotics to inhabitants of the Sunyani Municipality was very high. It is hence more likely for these drugs to be found at home. For solid, liquid, and semisolid dosages of medications, the predominant disposal routes were through the assistance of the Food and Drugs Authority. However, a notable percentage (about 30%) of respondents also admitted that disposal into garbage cans eventually ended up on dumpsites. Studies conducted in Ireland [25] and Afghanistan [20] have shown that OTCMs and pharmacies predominately dispose of pharmaceutical wastes into dump cans. Dumping pharmaceuticals into dump cans is an inappropriate disposal pattern. Both pharmacies and individuals in the Sunyani Municipality also disposed of unused and expired drugs inappropriately by leaving them in dump cans, similar to practices observed in Galway, Ireland, and Kabul, Afghanistan. Some of these pharmacies liaised with the FDA to dispose of unused and expired drugs. Generally, the respondents agreed largely that take-back programs back to wholesale sellers would be highly inconvenient due to the cost of transportation of products.

Furthermore, the respondents agreed on the need for a national medicine disposal scheme available to all pharmacies across the country to help them dispose of unused and expired drugs. For this scheme, respondents suggested that it should be run by the FDA and pharmaceutical companies because of their broad knowledge and expertise in the area. The respondent from the pharmacies was concerned about the environment. This was mainly due to their high literacy level. The Kabul study also confirmed that a high literacy level had a positive correlation with the concern of the respondents towards the environment, drug use and purchase, and drug disposal [20].

In this study, respondents agreed to prevent water pollution even when there was no evidence, indicating that they cared deeply about environmental problems that were not necessarily about their health or property. Respondents were also well informed of the environmental issues associated with the presence of pharmaceuticals. They, however, disagreed primarily on contributions to improve sewage treatment, mainly due to lack of trust in governance in the country. Gender and age played insignificant roles in the responses to questions from this survey.

Due to the COVID-19 restrictions that were imposed in the area during the study, only a limited number of households could be accessed. When access was given, only one participant was chosen to represent each household, and hence, the disposal patterns practised by other household members could not be examined. Notwithstanding, the study adds information to an area of little knowledge. For the first time in the Sunyani Municipality, there are baseline data on the disposal patterns of unused and expired medicines.

## 5. Conclusion

Most unused and expired drugs were disposed of by dumping them in dustbins or flushing them down the toilet. Although proper drug disposal methods such as the DUMP and take-back programs are available, their implementation does not cater to the entire Ghanaian population. There is very little awareness of such programs among households. These reasons have led to the indiscriminate dumping of analgesics and antibiotics in the environment and the consequent occurrence of these pollutants within the area. This may be the same for some regions within the country as well. Hence, it is recommended that DUMP policies that include households, individuals, and manufacturing companies should be instituted and supported to reduce the inappropriate disposal of unused and expired medications. Waste treatment methods such as waste segregation must be employed to separate unsafe products from general waste, such as unused and expired drugs. Therefore, appropriate drug disposal regulatory bodies must be deployed to ensure the safe disposal of unused and expired medicines within the communities.

## Figures and Tables

**Table 1:** Demographic data of household and pharmacy respondents in the Sunyani Municipality.

	Number of mentions	Percentage
Household category (*n* *=* 400)		
Gender		
Male	218	45.5
Female	182	54.5
Age (years)		
15–30	286	71.5
30–45	80	20.0
Above 45	34	8.5
Educational level		
Primary	17	4.3
JSS/JHS	137	34.3
SSS/SHS	118	29.5
Tertiary	10	27.3
No formal education	19	4.8

Pharmacy category (*n* *=* 35)		
Gender		
Male	17	48.6
Female	18	51.4
Age (years)		
15–30	19	54.3
30–45	5	14.3
Above 45	11	31.4
Educational level		
Primary	0	0
JSS/JHS	12	22.9
SSS/SHS	15	34.3
Tertiary	19	42.9
Position		
Pharmacist	7	20.0
Technician	5	14.3
Pharmacy assistant	5	14.3
Over-the-counter drug seller	12	34.3
Pharmacy store manager	6	17.1

JSS, junior secondary school; JHS, junior high school; SSS, senior secondary school; SHS, senior high school.

**Table 2:** A summary of household respondents' views on the impact of pharmaceutical residues on their external environment.

Question	Response (%)				
	Strongly agree	Disagree somewhat	Agree somewhat	Neither agree nor disagree	Strongly disagree
Households					
Taking my unused or expired medication to the pharmacy for disposal would be inconvenient?	39.8	22.0	13.5	8.0	16.8
We should only spend money on environmental problems that affect human health, not on problems that only affect other species or ecosystems.	31.0	17.3	7.5	12.3	31.8
We should try to prevent all water pollution, even if we have no evidence that a pollutant will harm humans or ecosystem health.	74.3	15.0	5.5	3.5	1.8
If an environmental problem does not affect my health or my property, I do not care about it	18.8	15.5	8.0	7.5	50.3

Pharmacies and over-the-counter medication shops					
We should only spend money on environmental problems that affect human health, not on problems that only affect other species or ecosystems.	28.6	17.1	5.7	5.7	42.9
We should try to prevent all water pollution, even if we have no evidence that a pollutant will harm human or ecosystem health.	60.0	11.4	11.4	2.9	14.3
If an environmental problem does not affect my health or my property, I do not care about it.	20.0	5.7	8.6	2.9	62.9
Taking unused or expired medication to the FDA or wholesalers for disposal is inconvenient?	42.9	17.1	14.3	8.6	14.3

## Data Availability

The data generated or analyzed during this study are included within the article.

## Supplementary Materials

Supplementary Materials
*Appendix 1.* Questions posed to pharmacy respondents. *Appendix 2.* Questions posed to household respondents.

## References

[1] Monteiro S. C., Boxall A. B. A. (2010). Occurrence and fate of human pharmaceuticals in the environment. *Reviews of Environmental Contamination & Toxicology*.

[2] Wang Y., Wang X., Li M., Dong J., Sun C., Chen G. (2018). Removal of pharmaceutical and personal care products (PPCPs) from municipal waste water with integrated membrane systems, MBR-RO/NF. *International Journal of Environmental Research and Public Health*.

[3] Kim J.-W., Jang H. S., Kim J. G. (2009). Occurrence of pharmaceutical and personal care products (PPCPs) in surface water from mankyung river, south Korea. *Journal of Health Science*.

[4] Gyesi J. N., Nyaaba B. A., Darko G. (2022). Occurrence of pharmaceutical residues and antibiotic-resistant bacteria in water and sediments from major reservoirs (owabi and barekese dams) in Ghana. *Journal of Chemistry*.

[5] Bexfield L. M., Toccalino P. L., Belitz K., Foreman W. T., Furlong E. T. (2019). Hormones and pharmaceuticals in groundwater used as a source of drinking water across the United States. *Environmental Science and Technology*.

[6] Botero-Coy A. M., Martinez-Pachon D., Boix C. (2018). An investigation into the occurrence and removal of pharmaceuticals in colombian wastewater. *Science of the Total Environment*.

[7] Reis E. O., Foureaux A. F. S., Rodrigues J. S. (2019). Occurrence, removal and seasonal variation of pharmaceuticals in brasilian drinking water treatment plants. *Environmental Pollution*.

[8] Darko G., Borquaye L. S., Acheampong A., Oppong K. (2017). Veterinary antibiotics in dairy products from Kumasi, Ghana. *Cogent Chemistry*.

[9] Mingle C. L., Darko G., Borquaye L. S., Asare-Donkor N. K., Woode E., Koranteng F. (2021). Veterinary drug residues in beef, chicken, and egg from Ghana. *Chemistry Africa*.

[10] Gros M., Mas-Pla J., Boy-Roura M., Geli I., Domingo F., Petrović M. (2019). Veterinary pharmaceuticals and antibiotics in manure and slurry and their fate in amended agricultural soils: findings from an experimental field site (baix empordà, NE Catalonia). *Science of the Total Environment*.

[11] Borquaye L. S., Ekuadzi E., Darko G. (2019). Occurrence of antibiotics and antibiotic-resistant bacteria in landfill sites in Kumasi, Ghana. *Journal of Chemistry*.

[12] de García S. O., García-Encina P., Irusta-Mata R. (2011). Environmental risk assessment (ERA) of pharmaceuticals and personal care products (PPCPs) using ecotoxicity tests. *Ecotoxicology*.

[13] Cooper E. R., Siewicki T. C., Phillips K. (2008). Preliminary risk assessment database and risk ranking of pharmaceuticals in the environment. *Science of the Total Environment*.

[14] Osei-Djarbeng S. N., Larbi G. O., Abdul-Rahman R., Osei-Asante S., Owusu-Antwi R. (2015). Household acquisition of medicines and disposal of expired and unused medicines at two suburbs (bohyen and kaase) in Kumasi-Ghana. *Pharma Innovation*.

[15] Glassmeyer S. T., Hinchey E. K., Boehme S. E. (2009). Disposal practices for unwanted residential medications in the United States. *Environment International*.

[16] Ghana National Drugs Programme (GNDP) (2004). Ghana national drug policy. *Accra: Ministry of Health*.

[17] Tong A., Peake B., Braund R. (2011). Disposal practices for unused medications in New Zealand community pharmacies. *Journal of Primary Health Care*.

[18] Poku M. K., Seidu O., Fayorsey C. K. (2013). *2010 Population and Housing Census, Regional Analytical Report - Brong Ahafo*.

[19] Ayele Y., Mamu M. (2018). Assessment of knowledge, attitude and practice towards disposal of unused and expired pharmaceuticals among community in Harar city, Eastern Ethiopia. *Journal of Pharmaceutical Policy and Practice*.

[20] Bashaar M., Thawani V., Hassali M. A., Saleem F. (2017). Disposal practices of unused and expired pharmaceuticals among general public in Kabul. *BMC Public Health*.

[21] Aboagye V. S., Kyei K. A. (2014). Disposal of leftover drugs in Ghana. *Asian Journal of Pharmaceutical Research*.

[22] Abruquah A. A., Drewry J. A., Ampratwum F. T. (2014). What happens to unused, expired and unwanted medications? a survey of a community-based medication disposal practices. *International Journal of Development and Sustainability*.

[23] Khan U., Bloom R. A., Nicell J. A., Laurenson J. P. (2017). Risks associated with the environmental release of pharmaceuticals on the U.S. food and drug administration flush list. *Science of the Total Environment*.

[24] Borquaye L. S. (2018). Disposal of unused and expired medicines in Ghana. *West African Journal of Pharmacy*.

[25] Vellinga A., Cormican S., Driscoll J., Furey M., O’Sullivan M., Cormican M. (2014). Public practice regarding disposal of unused medicines in Ireland. *Science of the Total Environment*.

