# Supplementary material for: Disposal of Unused and Expired Medicines within the Sunyani Municipality of Ghana: A Cross-Sectional Survey
Source: J Environ Public Health. 2022 May 26;2022:6113346. doi: 10.1155/2022/6113346 (PMC9162851; doi:10.1155/2022/6113346)
Supplement: Supplementary Materials — Appendix 1. Questions posed to pharmacy respondents. Appendix 2. Questions posed to household respondents. [file 6113346.f1.docx]

**Disposal of Unused and Expired Medicines Within the Sunyani Municipality of Ghana: a Cross-Sectional Survey**

Ivy Anima Amoabeng^1^, Bernice Araba Otoo^1^, Godfred Darko^1^ and Lawrence Sheringham Borquaye^1,2*^

^1^Department of Chemistry, Kwame Nkrumah University of Science and Technology, Kumasi-Ghana

^2^Central Laboratory, Kwame Nkrumah University of Science and Technology, Kumasi-Ghana

**SUPPLEMENTARY DATA**

**Appendix 1: Questions posed to pharmacy respondents**

| Questions | Response options |
| --- | --- |
| Are you a male or a female? | Male  Female |
| Could you tell us your age? | 18- 30yrs  31-45yrs  above 45yrs |
| What is your highest level of education? | Primary  JSS/JHS  Secondary School  Tertiary |
| How long have you practiced? | Over 40 years  31 – 40 years  21 – 30 years  11 – 20 years  Less than 10 years |
| What is your qualification? | Technician  Pharmacist  Over the counter drug seller  Pharmacy Assistant  Pharmacy store manager |
| Which of these prescriptions drugs do you vend out often? | Antimalarial  Anti-hypertension  Pain medication  Heart medication  Antibiotics |
| How do you discard expired or unused drugs;   1. Solid (tablets, capsules, etc. 2. Liquid (e.g. suspensions, elixirs) 3. Semi-solids (e.g. ointments, creams, etc.) | By dumping medicines in the garbage before disposal in landfill  By heat destruction  By flushing down the sink  Through the assistance of the FDA  Taken back to pharmaceutical wholesalers  Others |
| Does the FDA provide receipts when they assist you with the disposal of expired or unused drugs? | Yes  No |
| 1. We should disburse money only on environmental challenges that impact human health, not on challenges that impact other species or the natural environment. 2. We should try to avoid all water contaminations, even if we have no proof that a contaminate will distress humans or the ecosystem. 3. If an environmental challenge doesn’t impact my health or my property, I am not bothered. | Agree strongly  Agree somewhat  Neither agree or disagree  Disagree somewhat  Strongly disagree |
| Before reading this questionnaire, were you conscious that the contamination of streams and rivers by pharmaceuticals was an issue of environmental concern? | Yes  No |
| Will you be open to making payments to enhance sewage treatment so that streams and rivers' contamination by pharmaceuticals could be minimized? | Yes  No |

**Appendix 2: Questions posed to household respondents**

| Questions | Response options |
| --- | --- |
| Are you a male or a female? | Male  Female |
| Could you tell us your age? | 15- 30yrs  31-45yrs  above 45yrs |
| What is your highest level of education? | Primary  JSS/JHS  Secondary School  Tertiary  No formal education |
| Do you have medicines (tablets or syrups) in your home? | Yes  No  Don’t know |
| Do you have any antibiotics at home? | Yes  No  Don’t know |
| Do you have any analgesics at home? | Yes  No  Don’t know |
| Do you have any leftover, unused or unwanted prescription medications in your house at present? | Yes  No |
| How often do you dispose of unused or expired medication?   1. both prescription and non-prescription drugs 2. herbal medication | Weekly  Yearly  When I have no use  Never had unused or expired medications |
| How do you dispose of your unused or expired medication? | By placing medicines in the garbage before disposal in landfill  By incineration (or other forms of heat destruction)  By flushing down the sink  Through the assistance of the FDA  Taken back to pharmaceutical wholesalers  Others |
| Before reading this questionnaire, were you aware that you could return your unused medications to the pharmacy for proper disposal? | Yes  No |
| Which of the following prescription drugs have you disposed of? | Antimalarial  Antibiotics  Pain medication  Anti-hypertensives  Heart medication |
| Which of the following non - prescription drugs (aspirin, ibuprofen) do you dispose of | Pain medication  Antibacterial hand wash soaps  Herbal medications  Antimalarial  Cough & cold medications  Anti-histamines  Other |
| How would you rank the following 5 items in terms of how important they are to you? Rank the items from 1 to 5, with 1 being the most important and 5 the least important | Human Health  Transportation  Education  Environment  Jobs |
| Before reading this questionnaire, were you aware that the pollution of rivers and streams by pharmaceuticals was an environmental concern? | Yes  No |
| 1. Taking my leftover and expired medication back to drug dispensary shops for disposal would be a burden? 2. We should disburse money only on environmental challenges that impact human health, not on challenges that impact other species or the natural environment. 3. We should try to avoid all water contaminations, even if we have no proof that a contaminate will distress humans or the ecosystem. 4. If an environmental challenge doesn’t impact my health or my property, I am not bothered. | Agree strongly  Agree somewhat  Neither agree or disagree  Disagree somewhat  Strongly disagree |
| Will you be willing to pay to improve sewage treatment so that river and stream pollution by pharmaceuticals could be reduced? | Yes  No |
| If a drug you take in were dreadful for the environment, would you be happy to change it to a more environmentally safe drug? | Probably  Definitely  Not sure  Probably not  No |
